# New‐onset vitiligo following COVID‐19 disease

**DOI:** 10.1002/ski2.86

**Published:** 2022-01-28

**Authors:** A. Herzum, C. Micalizzi, M. F. Molle, A. Parodi

**Affiliations:** ^1^ Department of Dermatology Di.S.Sal. San Martino Polyclinic Hospital IRCCS University of Genoa Genoa Italy

## Abstract

**Background:**

Coronavirus disease 2019 (COVID‐19) disease and vaccines have been associated to various skin reactions, which are mostly similar amongst them. New onset of vitiligo and hypopigmentations have been described following COVID‐19 vaccination, but never after COVID‐19 infection.

**Objectives:**

We present the case of a 45‐year‐old woman, who developed vitiligo 2 weeks after COVID‐19 disease. Skin lesions stabilized after 1 month of initial spreading.

**Results:**

Vitiligo is a relatively common acquired pigmentary disorder, possibly caused by a T CD8+ cell‐mediated autoimmune process, which may be enhanced after the immune activation of COVID‐19 disease. Molecular mimicry and bystander activation have been advocated as possible pathogenic mechanisms of vitiligo after COVID‐19 vaccination. The same mechanisms may also be involved as possible vitiligo triggers during COVID‐19 disease.

**Conclusions:**

Clinicians should be aware of this possible autoimmune cutaneous reaction to COVID‐19 disease.

## Graphical abstract

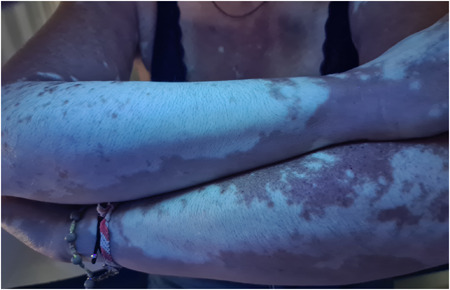

Dear Editor,

Coronavirus disease 2019 (COVID‐19) has been associated to numerous cutaneous manifestations, including morbilliform and urticarial rashes, vesicular eruptions, purpuric, petechial, livedoid, and acral chilblain‐like lesions.^1^, ^2^ In addition, COVID‐19 vaccines can cause a variety of skin reactions, mostly similar to the ones reported after COVID‐19 infection.^3^, ^4^ New onset of vitiligo and hypopigmentations have been described following COVID‐19 vaccination.^5^, ^6^, ^7^, ^8^ However, vitiligo has never been described after COVID‐19 infection up to date.

A 45‐year‐old woman presented with sharply demarcated milky‐white macules on her limbs, face and trunk (Figure 1a). The first lesions had appeared in early springtime on her axillae, only 2 weeks after mildly symptomatic, serologically confirmed, COVID‐19 disease, in association with anosmia and ageusia. The patient had barely noticed the hypopigmented lesions at first, being more concerned about systemic COVID‐19 symptoms. However, during summertime, skin lesions had spread on her upper limbs, trunk, face, groin and lower limbs, becoming more and more visible, in contrast to the patient's tanned skin after sun exposure and the patient decided to seek for medical advice.

**FIGURE 1:**
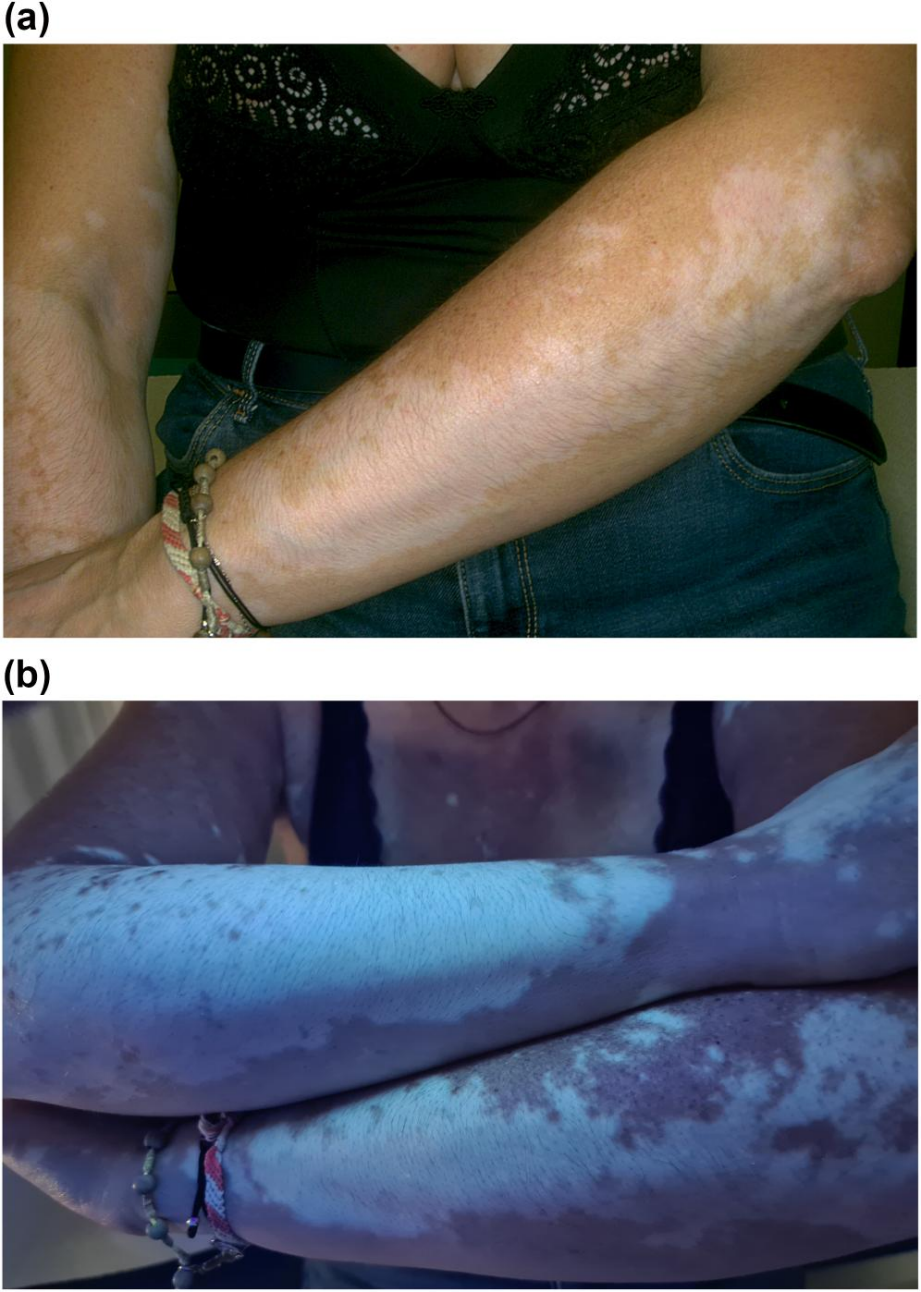
(a) Vitiligo of the arms: milky‐white round confluent macules. (b) Pathognomonic white fluorescence under the wood lamp of vitiligo macules of the arms

In late autumn, when the patient came to our attention, lesions had stabilized, but were still strongly evident, in contrast to the surrounding tanned skin. At clinical examination, the well‐defined hypopigmented macules were consistent with non‐segmental stable vitiligo. Also, wood lamp examination confirmed the diagnosis revealing characteristic white fluorescence (Figure 1b). Autoimmunity screenings including thyroid antibodies, anti‐nuclear antibodies, rheumatoid factor, as well as complete blood cell count and fasting blood glucose levels, were within normal ranges. The patient did not take any medications and her medical and family history were unremarkable. The patient will undergo narrow‐band UVB phototherapy to stimulate repigmentation. Written informed consent was obtained from the patient for publication of this case report and any accompanying images.

Vitiligo is an acquired pigmentary disorder affecting 0.5%–2% of the global population. It can be classified considering the clinical involvement as segmental (localized) or non‐segmental (generalized) and considering disease activity as stable or progressing.^7^, ^9^ The aetiopathogenesis of vitiligo is still debated, but there is evidence of a possible T CD8+ cell‐mediated autoimmune process, triggered by oxidative stress.^9^ Of note, immune activation during COVID‐19 disease might increase vitiligo disease activity through a shift towards adaptive type 1 immunity (CD8 T cells and IFNγ).^9^

Also, Pfizer‐BioNTech vaccine BNT162b2 (Cominarty) has already been linked to upregulation of Th1 response, causing increased levels of IL‐2, IFN‐γ and TNFα. These inflammatory cytokines have been associated to lichen planus reactivation and may also be involved in the pathogenesis of other autoimmune skin diseases, such as vitiligo.^10^, ^11^

Up to date, new‐onset vitiligo has been described several days after the first dose of mRNA‐1273 (Moderna) COVID‐19 vaccination, with progression after the second dose, in an otherwise healthy 61‐year‐old woman. Also, new‐onset vitiligo was reported 1 week after the first dose of Pfizer‐BioNTech vaccine BNT162b2 (Cominarty) COVID‐19 vaccine, in an otherwise healthy 33‐year‐old woman and in a 58‐year‐old man with ulcerative colitis.^5^, ^6^, ^7^ Similarly, aspecific hypopigmentations have been described in two patients as first‐dose reactions to mRNA‐1273 (Moderna) COVID‐19 vaccination or Pfizer‐BioNTech vaccine BNT162b2 (Cominarty) (Table 1).^8^

**TABLE 1:** Literature reported cases of vitiligo and hypopigmentation after COVID‐19 vaccination

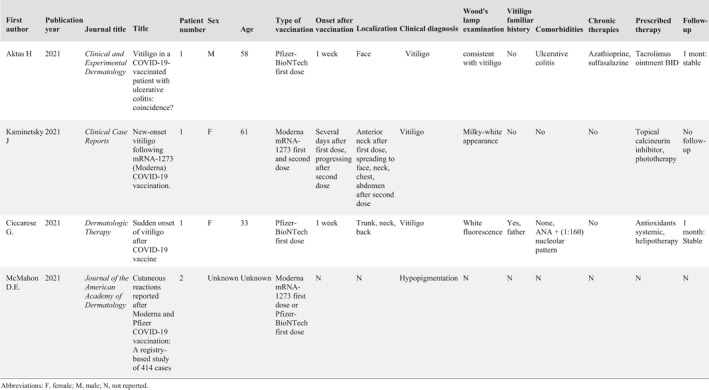

Regarding the onset of vitiligo after COVID‐19 vaccination, molecular mimicry and bystander activation have been advocated as possible pathogenic mechanisms.^7^ The same mechanisms of molecular mimicry, defined as cross‐reactivity to viral antigens, or of bystander activation, defined as viral‐induced release of sequestered self‐antigens, may be involved as possible vitiligo triggers during COVID‐19 disease itself, inducing a pathogen‐specific immune response directed also against host's melanocytes. Indeed, many cutaneous reactions observed after COVID‐19 vaccination mimic the skin lesions of SARS‐CoV‐2 infection itself, suggesting that both similar skin manifestations are more likely to be caused by analogous immune responses, rather than by the virus itself.^8^ Indeed, molecular mimicry‐induced autoimmunity has been described between SARS‐CoV‐2 antigens and host–tissue components.^4^ ^,^ ^12^

Noteworthy, a case of bullous pemphigoid arising after COVID‐19 infection has already been described after an initial acral vesicular eruption. It has been hypothesized that prolonged skin inflammation during initial viral exanthem may have damaged the basement membrane, rendering it susceptible to the host's immune recognition with subsequent development of autoantibodies.^12^

However, it must be considered that the occurrence of vitiligo after COVID‐19 infection might just be coincidental. Indeed, vitiligo has a high global prevalence (0.5%–2%) and has never been associated to COVID‐19 disease before.^7^, ^9^ However, the frequency of autoimmune phenomena occurring after COVID‐19 disease and the short time between vitiligo occurrence and COVID‐19 disease in our patient suggest a possible association of the two entities. Clinicians should be aware of this possible autoimmune cutaneous reaction while further reports and studies are necessary to demonstrate if a causal relationship between COVID‐19 infection and vitiligo exists.

## CONFLICT OF INTEREST

The authors declare no conflict of interests.

## AUTHOR CONTRIBUTIONS

**A. Herzum:** Conceptualization; Data curation; Formal analysis; Investigation; Methodology; Project administration; Supervision; Validation; Visualization; Writing – original draft; Writing – review & editing. **C. Micalizzi:** Conceptualization; Data curation; Formal analysis; Investigation; Methodology; Project administration; Supervision; Validation; Visualization; Writing – original draft; Writing – review & editing. **M. F. Molle:** Conceptualization; Data curation; Formal analysis; Investigation; Methodology; Project administration; Supervision; Validation; Visualization; Writing – original draft; Writing – review & editing. **A. Parodi:** Conceptualization; Data curation; Formal analysis; Investigation; Methodology; Project administration; Supervision; Validation; Visualization; Writing – original draft; Writing – review & editing.

## Data Availability

The data that support the findings of this study are available on request from the corresponding author.
